# Bluetongue virus infection induces aberrant mitosis in mammalian cells

**DOI:** 10.1186/1743-422X-10-319

**Published:** 2013-10-28

**Authors:** Andrew E Shaw, Anke Brüning-Richardson, Ewan E Morrison, Jacquelyn Bond, Jennifer Simpson, Natalie Ross-Smith, Oya Alpar, Peter PC Mertens, Paul Monaghan

**Affiliations:** 1 The Pirbright Institute, Pirbright, Woking GU24 0NF, UK; 2 Section of Oncology and Clinical Research, Leeds Institute of Cancer and Pathology, Leeds University, Leeds LS9 7TF, UK; 3 Section of Ophthalmology and Neuroscience, Leeds Institute of Biomedical and Clinical Sciences, Leeds University, Leeds LS9 7TF, UK; 4 School of Pharmacy, University of London, London WC1N 1AX, UK; 5 Faculty of Pharmacy, Istanbul Kemerburgaz University, Bagcilar, Istanbul, Turkey; 6 Australian Animal Health Laboratory, Geelong VIC 3220, Australia; 7 Current address: MRC-University of Glasgow Centre for Virus Research, 464 Bearsden Road, Glasgow G61 1QH, UK

**Keywords:** Bluetongue, Virus, Mitosis, Non-structural

## Abstract

**Background:**

Bluetongue virus (BTV) is an arbovirus that is responsible for ‘bluetongue’, an economically important disease of livestock. Although BTV is well characterised at the protein level, less is known regarding its interaction with host cells. During studies of virus inclusion body formation we observed what appeared to be a large proportion of cells in mitosis. Although the modulation of the cell cycle is well established for many viruses, this was a novel observation for BTV. We therefore undertook a study to reveal in more depth the impact of BTV upon cell division.

**Methods:**

We used a confocal microscopy approach to investigate the localisation of BTV proteins in a cellular context with their respective position relative to cellular proteins. In addition, to quantitatively assess the frequency of aberrant mitosis induction by the viral non-structural protein (NS) 2 we utilised live cell imaging to monitor HeLa-mCherry tubulin cells transfected with a plasmid expressing NS2.

**Results:**

Our data showed that these ‘aberrant mitoses’ can be induced in multiple cell types and by different strains of BTV. Further study confirmed multiplication of the centrosomes, each resulting in a separate mitotic spindle during mitosis. Interestingly, the BTV NS1 protein was strongly localised to the centrosomal regions. In a separate, yet related observation, the BTV NS2 protein was co-localised with the condensed chromosomes to a region suggestive of the kinetochore. Live cell imaging revealed that expression of an EGFP-NS2 fusion protein in HeLa-mCherry tubulin cells also results in mitotic defects.

**Conclusions:**

We hypothesise that NS2 is a microtubule cargo protein that may inadvertently disrupt the interaction of microtubule tips with the kinetochores during mitosis. Furthermore, the BTV NS1 protein was distinctly localised to a region encompassing the centrosome and may therefore be, at least in part, responsible for the disruption of the centrosome as observed in BTV infected mammalian cells.

## Background

Bluetongue virus (BTV) is an arbovirus that is transmitted between its ruminant hosts by species of *Culicoides* biting midge. The infection of ruminants with BTV can result in bluetongue (BT), an economically important disease of livestock. BTV is the type species of the genus *Orbivirus*, family *Reoviridae*, with a genome composed of ten segments of linear dsRNA (Seg-1 to Seg-10). Each genome segment encodes a distinct protein, with the exception of Seg-9 which encodes two proteins: the viral helicase VP6 and a recently discovered non-structural protein NS4 in the +1 reading frame [1,2]. The VP6 and NS3 proteins encoded by Seg-9 and Seg-10 can also be translated from different initiation codons near the upstream terminus of the mRNA, leading to related but different length protein-products (NS3/NS3A or VP6/VP6A) [3,4].

The BTV particle is arranged as three concentric shells, composed of the structural proteins VP3 and VP7 (making up the virus inner-core and outer-core layers respectively), with an additional outer capsid layer comprising VP2 and VP5. The dsRNA segments are encased within the central space of the core and are associated with transcriptase complexes made up of VP1 (RNA dependent RNA polymerase), VP4 (capping enzyme) and VP6 (helicase). Non-structural protein 1 (NS1) is encoded by Seg-5 and is an abundantly expressed protein that has been associated with cytopathogenicity and the translation of viral mRNAs [5,6]. NS3/3A are glycoproteins involved in virus egress and release via interactions with both VP2 and host cell factors [7-11]. Recently, NS3/3A has also been implicated in abrogating the type I interferon response [12]. NS4 has a nucleolar localisation and has been demonstrated to benefit virus replication in the presence of type I interferon [1].

NS2, which is encoded by Seg-8, is also highly expressed in BTV infected cells and is a major component of the viral inclusion bodies (VIBs) that are characteristic of BTV and other orbivirus infections [13]. VIBs, which are rich in ssRNA and all of the viral core proteins, are thought to be the major site of virus replication and assembly [13,14]. It has also been shown that NS2 binds to ssRNA via the N-terminus, and is specific for BTV (+)RNA, possibly via RNA secondary structures characteristic for each BTV segment [15-19]. In addition, NS2 is the only orbivirus protein that is phosphorylated, a process mediated by the cellular kinase CK2 [20,21].

Structural studies of NS2 indicate intermolecular interactions and suggest that it forms helical oligomers that have enhanced stability when interacting with RNA [21-23]. The carboxy terminus of the protein appears to play a role in the assembly of oligomers and therefore the aggregation of NS2 into larger structures [22,23]. Phosphorylation reduces the affinity of NS2 for ssRNA and appears to increase the number of VIBs that form near the nucleus, in contrast to the diffuse NS2 staining pattern of the dephosphorylated protein [21]. The dynamic nature of NS2 is emphasised by its ability to hydrolyse ATP and GTP, and the importance of the role played by NS2 is highlighted by the expression of functional homologs by other members of the family *Reoviridae* (Rotavirus and Reovirus) [24,25].

Despite the fundamental importance of NS2 and VIBs in the BTV lifecycle, remarkably little is known regarding the processes of VIB formation. NS2 is expressed early during infection and appears first as dots throughout the cytoplasm before agglomerating into mature VIBs. Whilst investigating the formation of VIBs, we observed an excess of aberrant mitoses in infected cells. Using a confocal and live cell imaging approach we characterised in more detail the induction of aberrant mitoses by BTV. We observed NS1 clustering around the centrosome and a previously undescribed interaction of NS2 with the centromeres of chromosomes.

## Results

### Aberrant cell division during BTV infection

During initial studies of NS2 interaction with microtubules in BHK-21 cells in the context of a BTV infection, we observed a substantial number of cell divisions that appeared abnormal. The most conspicuous feature of BTV infected cell cultures examined by confocal microscopy was the large number of rounded cells, apparently arrested in mitosis.

**Figure 1:**
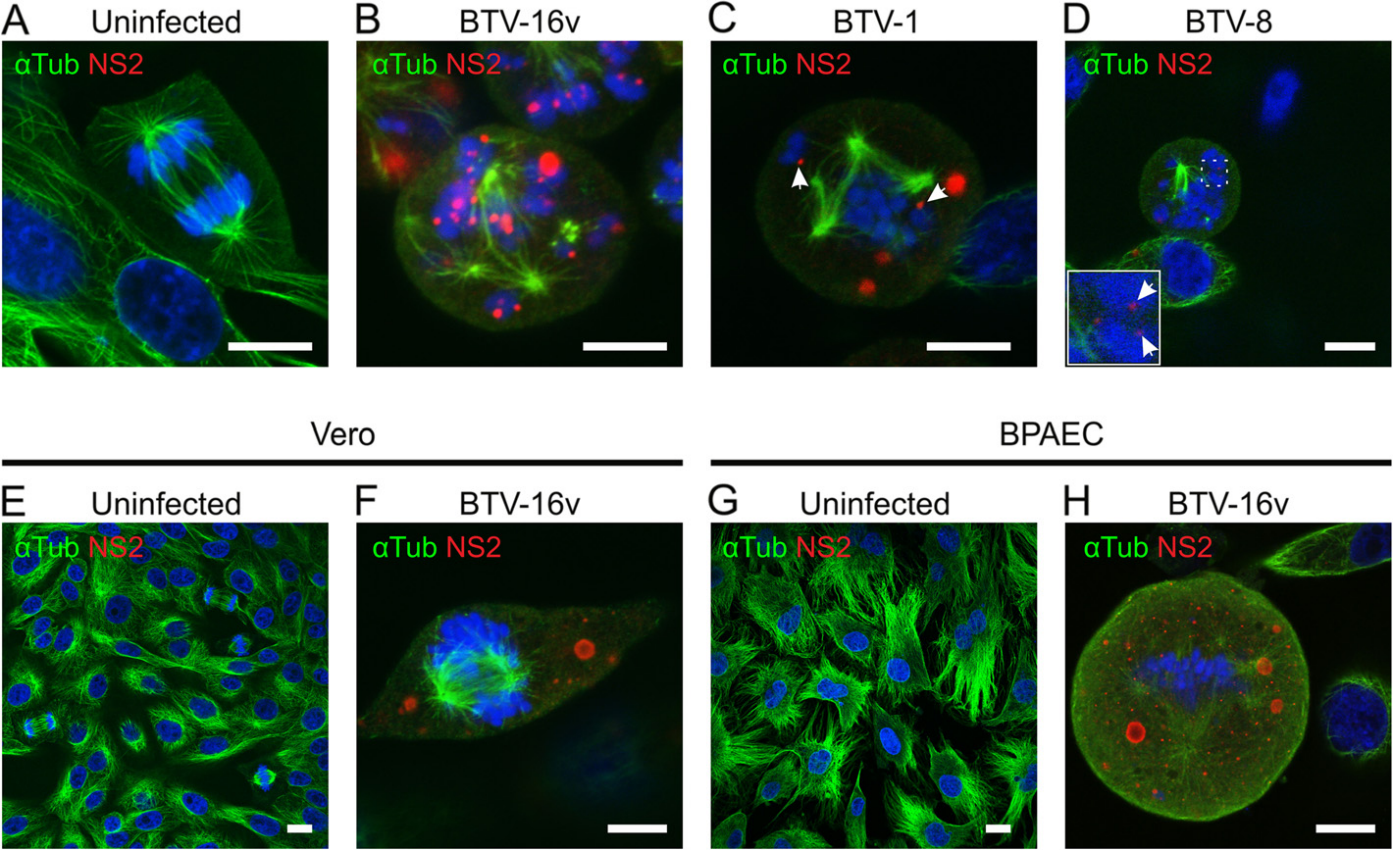
**BTV induces aberrant mitosis in cultured mammalian cells.** Cells were cultured in the presence of growth medium containing 10% serum and either infected or mock infected with BTV. At 16–24 hours post infection cells were fixed with paraformaldehyde and prepared for confocal immunofluorescence microscopy as described in the Materials and Methods. **(A)** Uninfected BHK-21 cells showed highly organised and symmetrical microtubule spindles. **(B)** In contrast, BTV-16v infected mitotic cells had multiple, disorganised and asymmetric spindles (alpha tubulin labelling in green) that were disassociated from the condensed chromosomes (blue). **(C)** and **(D)** BTV-1 and BTV-8 were also able to induce aberrant mitosis in BHK-21 cells, with NS2 (red) associated with the chromosomes. Vero cells and bovine pulmonary aortic endothelial (BPAEC) cells infected with BTV-16v **(F)** and **(H)** also showed abnormal mitotic events compared to the uninfected controls **(E)** and **(G)**. Scale bar = 10 μm.

To further investigate these phenomena, infected and uninfected BHK-21 cells were cultured in the presence of 10% fetal bovine serum (FBS), fixed at 16 h post infection (PI), then immunolabeled for NS2 and α-tubulin (which labels microtubules forming the mitotic spindle). Uninfected cells showed a normal pattern of microtubule distribution, with mitotic cells containing a spindle and, during metaphase, a robust metaphase plate. During anaphase, the chromosomes separated normally and migrated towards the spindle poles (Figure 1A). In contrast, immunolabeling of BTV infected cells revealed a disorganised pattern of α-tubulin distribution, often with multiple spindles and condensed chromosomes that were disorganised and not attached to a mitotic spindle (Figure 1B-D). BTV NS2 protein was also detected associated with the condensed chromosomes (Figure 1B-D).

### BTV-induced aberrant mitosis is independent of both virus and cell type

BTVs can be broadly divided into topotypes according to their geographic origin, either of eastern (Asia and Australasia), or western (African and New World) origin [26]. The arrest of mitosis caused by BTV infection was initially observed using the eastern topotype BTV-16 vaccine strain (BTV-16v). This BTV strain has been highly passaged in BHK-21 cells and may therefore have acquired particular characteristics for replication in these cells. BHK-21 cells infected with the reference strain of BTV-1 (Figure 1C) or the UK field strain of BTV-8 (UKG2008/34, Figure 1D), both of which are western topotype strains, also showed evidence of abnormal cell divisions, with multiple spindles and NS2 associated with the chromosomes in a manner reminiscent of that observed with BTV-16v. This indicates that these phenomena are not specific to BTV-16v and are not restricted by topotype, or simply the result of adaptation to cell cultures.

BHK-21 cells are highly permissive for BTV and there is evidence in the literature that the cytopathology induced by BTV varies according to cell type [27,28]. We therefore infected Vero and bovine pulmonary aortic endothelial (BPAEC) cells to determine whether mitosis in other cells was affected in a similar manner. Uninfected Vero and BPAEC cells showed normal mitotic processes and patterns of α-tubulin labelling (Figure 1E and G). Although some of the infected Vero and BPAEC cells showed a normal mitotic pattern of chromosomes, there were also infected cells with multiple spindle poles and aberrantly distributed chromosomes (as previously observed in BHK-21 cells, Figure 1F). Similar results were obtained in infected BPAEC cells, with evidence of multiple spindle poles and apparently random distributions of condensed chromosomes (Figure 1H). These results demonstrate that the blockage of mitosis induced by BTV is cell line independent, although BHK-21 cells appeared to be the most susceptible. Overall, although present, the frequency of aberrant mitosis was reduced in the case of non-BHK cells and with viruses other than BTV-16v.

### Bluetongue virus disrupts the microtubule organising centre

**Figure 2:**
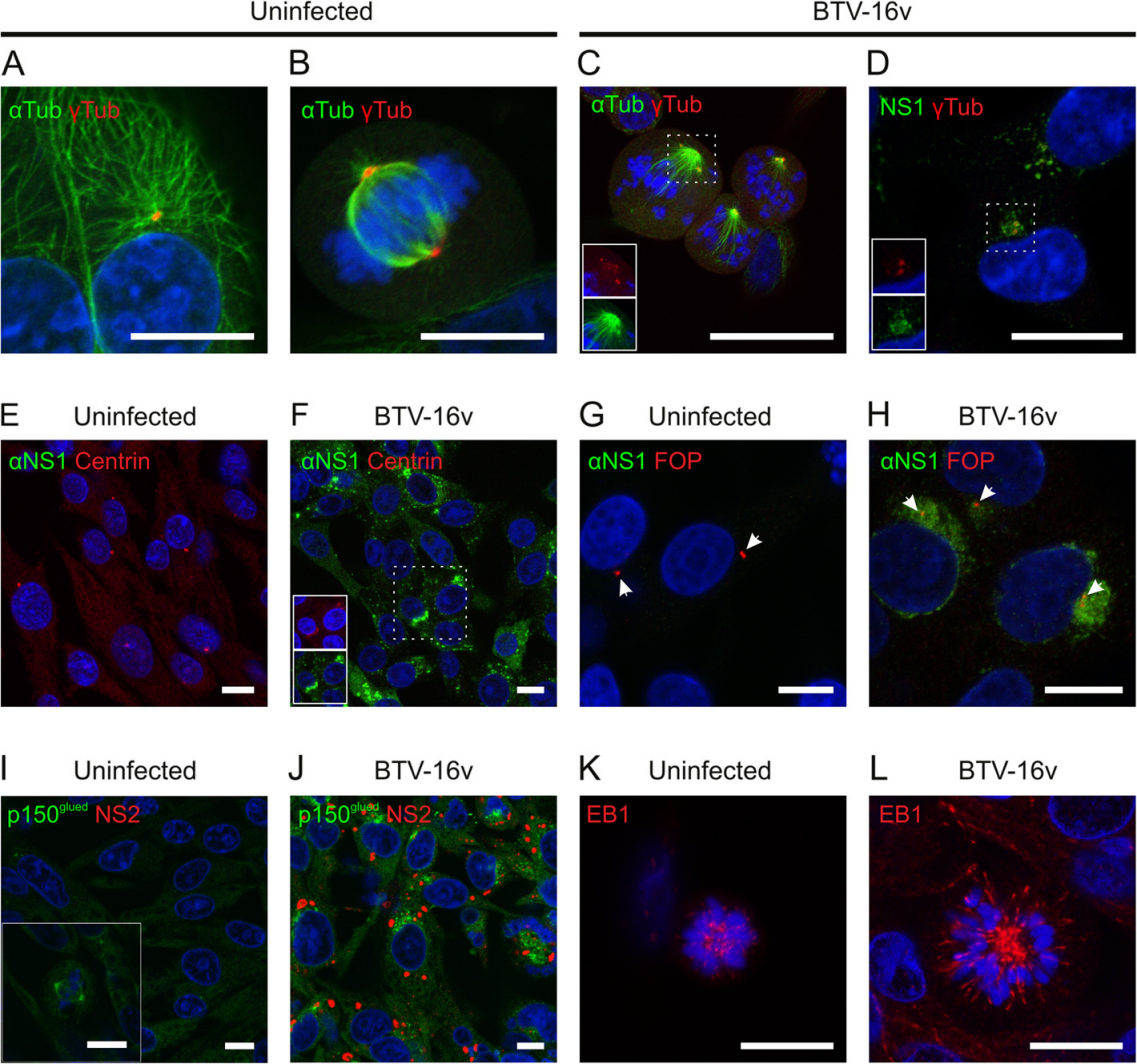
**BTV affects the microtubule organising centre (MTOC) and disrupts the interaction between condensed chromosomes and microtubules.** BTV-16v-infected or mock infected BHK-21 cells were fixed and assessed for microtubule organising centre (MTOC) integrity using confocal microscopy. **(A)** Uninfected cells in interphase showed a loose array of microtubules (alpha tubulin labelling, green) with a single MTOC (gamma tubulin labelling, red). **(B)** Uninfected but mitotic cells contained a single highly organised microtubule spindle with an MTOC at each pole, associated with the condensed chromosomes (blue) arranged perpendicular to the spindle. Mitotic cells infected with BTV-16v lacked organisation and symmetry in the spindle and the MTOC appeared to be disrupted **(C)**, with the MTOC also disrupted in infected cells in interphase **(D)**. Centrin labelling was altered, from a largely punctate form in uninfected cells **(E)** to a more dispersed distribution in infected cells **(F)**. NS1 (green) localised to the region of the MTOC. **(G,H)** In contrast, FOP (arrows) labelling remained punctate in both infected and uninfected cells. **(I)** In uninfected cells, p150^glued^ (green) exhibited a diffuse cytoplasmic distribution in interphase and a spindle-like formation in mitosis (insert), but in infected cells **(J)** p150^glued^ was more condensed and localised towards the nucleus in a region suggestive of the MTOC. **(K)** EB1 labelling of the growing tips of microtubules shows a close association with the condensed chromosomes in uninfected mitotic cells. **(L)** In contrast, EB1 labelling and the microtubule tips showed a wider distribution and less association with the condensed chromosomes in infected mitotic cells. Scale bar = 10 μm.

A conspicuous feature of BTV infected cells showing aberrant mitosis was the presence of asymmetric spindles and multiple spindle poles. The microtubule organising centre (MTOC) plays an essential role in the organisation and polymerisation of the microtubule network [29]. During interphase, the MTOC resides in a perinuclear location and anchors an array of microtubules (Figure 2A).

We hypothesised that the MTOC might be disrupted in infected cells, leading to disorganised spindles during mitosis. First, in order to determine whether each mitotic spindle pole contained a MTOC, BHK-21 cells were infected (or mock infected) with BTV-16v and immunolabeled for α-tubulin (microtubules) and γ-tubulin (MTOC). In uninfected mitotic cells, γ-tubulin was located in the expected position at the pole of each half spindle (Figure 2B). In contrast, although recognizable spindle structures were still present/being formed in BTV infected cells, microtubule and spindle organisation were conspicuously disrupted. Spindles also lacked the symmetry of those seen in uninfected cells and a monopolar configuration was frequently observed (Figure 2C). Furthermore, although the MTOC remained in a perinuclear location in infected cells, γ-tubulin labelling showed two adjacent dots, which often appeared ‘smudged’, rather than the punctuate form observed in uninfected cells (Figure 2C).

Previous TEM studies have shown that the NS1 protein clusters towards the nucleus, including around the centrosome, in BTV infected cells, (A. Hyatt and P. Monaghan, unpublished observations). To investigate if NS1 disrupted MTOCs in non-mitotic cells, BTV-16v infected BHK-21 cells were fixed and immunolabeled using antibodies to NS1 and γ-tubulin. Centrosomal γ-tubulin labelling was again disrupted in infected cells where it co-localised with NS1 labelling (Figure 2D).

These findings indicated that the centrosome was disrupted in BTV infected cells. We sought to confirm and extend this observation by examining the fate of other MTOC-associated proteins in infected cells. During the S-phase of each cell cycle the centrosome is duplicated to provide MTOCs for both daughter cells. Centrin plays a fundamental role in the biogenesis of centrosomes and can therefore be considered as a measure of centrosome health [30]. In uninfected cells, immunolabeling showed that centrin was present as a punctate dot adjacent to the nucleus, a typical location for the centrosome (Figure 2E). However, in infected cells centrin was either absent or dispersed (Figure 2F). The most intense accumulations of NS1 appeared to co-localise with the disrupted centrin, although aggregates of NS1 were also observed elsewhere within the cell (Figure 2F). We next investigated the effect of BTV infection on a panel of proteins associated with the centrosome or MT anchoring, including FOP, EB1 and p150^glued^. FOP is a centrosomal protein that is preferentially located towards the distal end of the mother centriole. Along with EB1 and p150^glued^, FOP helps anchor microtubules to the centrosome [31]. In uninfected interphase cells, FOP labelling was observed adjacent to the nucleus (Figure 2G). In contrast to the γ-tubulin and centrin labelling, FOP labelling remained punctate in infected cells (Figure 2H). In the majority of infected interphase cells, FOP labelling was observed as two dots adjacent to the nucleus (Figure 2H). Although in mitotic cells the number of FOP dots did not appear to alter, FOP remained localised to the spindle poles in a similar manner to the uninfected cells. Interestingly, aggregations of NS1 also co-localised with FOP (Figure 2H).

In addition to its role at the centrosome, p150^glued^ is also an integral component of the dynactin complex and is involved in directly binding to the intermediate chains of dynein [32]. Dynein performs critical roles in intracellular transport and in spindle organisation and function [33]. In uninfected cells immunolabeled for p150^glued^ and NS2, p150^glued^ was distributed throughout the cytoplasm (Figure 2I). In contrast, in BTV infected cells, p150^glued^ was dramatically redistributed into granular masses to one side of the nucleus, which previous data indicate may encompass the region of the centrosome (Figure 2J).

### Condensed chromosomes fail to attach to polymerising microtubules in infected cells

The immunolabeling patterns of microtubules and the MTOC/centrosome implied inherent defects in the formation and/or organisation of the mitotic spindle in infected cells. The EB1 protein family is a highly conserved group of microtubule associated proteins which specifically localise to the growing distal tip of microtubules [34]. EB1 microtubule end labelling can therefore be used to assess the dynamic polymerisation of the microtubules themselves.

The polymerisation of microtubules was assessed in BHK-21 cells showing aberrant mitosis at 24 hours post infection with BTV-16v using confocal microscopy. In terms of presence, there appeared to be no clear differences in the labelling of EB1 in infected and uninfected cells (Figure 2K-L). In uninfected mitotic cells, EB1 labelling appeared to be more focused and was localised to spindle poles and the growing tips of the mitotic spindle microtubules, and was usually restricted to a region within and closely associated with the condensed DNA forming the normal prometaphase rosette (Figure 2K). In BTV-16v infected cultures, cells showing aberrant mitosis with an abnormal distribution of condensed chromosomes also showed intense EB1 labelling at microtubule tips, indicating that microtubules were still capable of polymerising (Figure 2L). However, EB1 labelling of microtubule tips, was less concentrated in the vicinity of the condensed chromosomes, (Figure 2L). In infected cells the distribution of labelled-tips suggested that the microtubule ends were growing past the more loosely organised condensed chromosomes. This was reminiscent of results seen when the function of proteins that promote microtubule capture at kinetochores are disrupted, such as CLASP proteins [35], suggesting that microtubule-chromosome interaction via the kinetochore had been lost in BTV infected cells.

### NS2 alone can interact with microtubules

**Figure 3:**
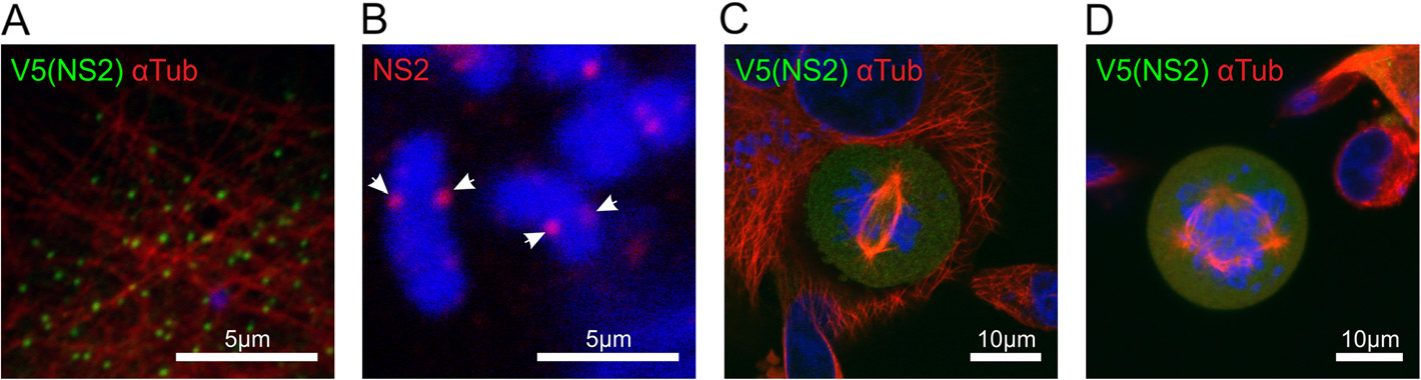
**BTV non-structural protein 2 interacts with microtubules and the condensed chromosomes, and can induce aberrant mitoses in the absence of other BTV proteins. (A)** Plasmid pNS2-V5 was transfected into uninfected BHK-21 cells. After 24 hours the cells were fixed and immunolabeled using an antibody targeting the V5 epitope (green). Microtubules were revealed using alpha tubulin labelling (red). Small aggregates of NS2 were closely associated with the microtubules, suggesting that other BTV proteins are not required for the interaction to occur. Scale bar = 5 μm. **(B)** In BTV-16v infected mitotic BHK-21 cells, NS2 (red - arrows) was observed (using z-stack and deconvolution analysis) associated with the condensed chromosomes (blue) in locations suggestive of the kinetochores. Scale bar = 5 μm. **(C)** and **(D)** Uninfected mitotic cells transfected with pNS2-V5 (green) exhibited similar aberrant mitoses to those observed during BTV infection, including chromosomes localised parallel to the microtubule spindle **(C)** and multiple spindles **(D)**. In these mitotic cells, NS2-V5 staining was diffuse, as opposed to the condensed VIB-like form seen in BTV infected cells. Scale bar = 10 μm.

NS2 can be detected early after infection and aggregates that resemble VIBs can be observed as early as four hours post infection [13,14]. Although it is possible to immunolabel for BTV NS2 and observe interactions with microtubules in infected cells, labelling NS2 in the context of an infection does not rule out the possibility that the association of NS2 with microtubules is indirect and mediated by another BTV protein. To investigate whether the observed association with microtubules is an intrinsic feature of NS2, we expressed the protein from a plasmid vector in transiently transfected cells. When BHK-21 cells were transiently transfected with pNS2-V5 and immunolabeled with a monoclonal antibody to the V5 epitope, small dots of NS2 were observed in close association with microtubules in interphase cells (Figure 3A).

### BTV NS2 localises to chromosomes in infected cells

One of the consistent observations made by confocal microscopy and immunolabeling of aberrant mitotic cells is the association of NS2 with the condensed chromosomes.

The attachment of chromosomes to the mitotic spindle via kinetochore-mediated capture of microtubule tips is a fundamental part of the cell division mechanism. The consistent association of NS2/VIBs with condensed chromosomes, combined with the importance of microtubules in cell division, prompted us to perform 3D stacks of NS2 immunolabeled images of infected cells undergoing aberrant mitosis. The resulting images showed NS2 was regularly observed at two symmetrical locations on the chromosomes that strongly suggest interactions with the kinetochores (Figure 3B). Combined with the data indicating an interaction between NS2 and the microtubules, these data together suggest that NS2 is either a ‘cargo-molecule’ for a microtubule motor protein and/or a direct interaction exists between NS2 and a cellular kinetochore protein.

The plasmid pNS2-V5 was transfected into uninfected BHK-21 cells and the fixed cells were immunolabeled for the V5 epitope. In each case where a cell was both transfected and in the process of cell division, the cell contained evidence of aberrant mitosis (Figure 3C-D). Multiple spindle poles were seen in these cells, as was chromosomal misalignment in metaphase cells (Figure 3C-D). NS2 labelling appeared evenly distributed throughout these dividing cells, with little if any evidence of VIB-like structures interacting with condensed chromosomes (Figure 3C-D). These data confirm that NS2 alone has the ability to reproduce major aspects of the aberrant mitotic phenotype observed in cells infected with BTV.

### BTV NS2 disrupts cell division in live cell imaging

**Figure 4:**
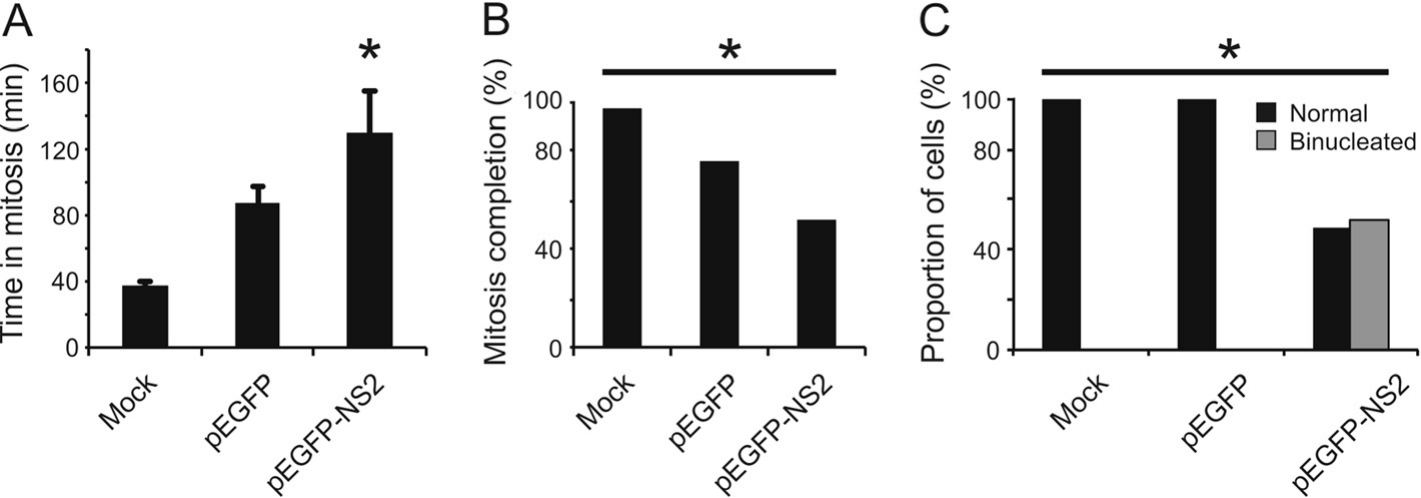
**Live cell imaging of HeLa-mCherry tubulin cells shows that transfection with EGFP-NS2 leads to prolonged mitosis and binucleation events.** HeLa-mCherry cells mock transfected or transfected with plasmids expressing either EGFP or an EGFP-NS2 fusion proteins were followed using live cell imaging as described in Materials and methods. **(A)** Cells transfected with pEGFP-NS2 spent a significantly longer time in mitosis than cells transfected with pEGFP alone, or untransfected cells (p = 0.038). **(B)** Cells expressing EGFP-NS2 showed the lowest level of completed mitosis (p < 0.0001). **(C)** In cells that successfully completed mitosis, only those transfected with EGFP-NS2 displayed binucleation events (p < 0.0001), while mock transfected cells, or cells expressing EGFP alone showed a normal mitosis outcome.

Cell division is a dynamic process and is therefore not entirely captured using fixed cell cultures. We therefore used live-cell fluorescence microscopy to further investigate BTV-induced perturbation of cell division. Plasmids expressing either EGFP-NS2 or EGFP alone were transiently transfected into HeLa-mCherry tubulin cells and observed using live cell imaging. Cells transfected with either plasmid spent a longer period of time in mitosis than untransfected cells (mock transfected cells 37. 6 ±1.8 min.; pEGFP 87.3 ±9.8 min.; pEGFP-NS2 131.7 ±23.5 min., Figure 4A). However, expression of EGFP-NS2 caused cells to spend a significantly longer period in mitosis relative to those expressing EGFP (Figure 4A, t-test, p = 0.038). Furthermore, significantly fewer cells expressing EGFP-NS2 completed mitosis relative to those expressing EGFP (Figure 4B, Chi-Square Test, p < 0.0001). Binucleation was also observed significantly more often in pEGFP-NS2 transfected cells than in those transfected with pEGFP, or in untransfected cells (Figure 4C, Chi-Square Test, p < 0.0001). In summary, expression of EGFP-NS2 resulted in increased rates of mitotic arrest, an extended period of time spent in mitosis, and increased the proportion of cell divisions that resulted in a binucleated cell.

## Discussion

During infection of mammalian cells, Bluetongue virus NS2 is translated and distributed at multiple points throughout the cytoplasm, before aggregating to form larger VIBs. The likelihood of such structures forming merely by diffusion is low and a mechanism must therefore exist to gather together the VIB components, of which NS2 is a primary constituent. Microtubules are critical for the shuttling of ‘cargo’ around the cell, including retrograde transport of cargo towards the nucleus [36], and previous work has revealed interactions between BTV and the cytoskeleton [37,38]. However, these initial experiments used extracted cytoskeletons and scanning electron microscopy. Transient-expression studies of NS2-V5 showed that it is able to associate with microtubules in the absence of the other BTV proteins. However, blocking of the C terminus with a V5 epitope tag precluded assembly into the larger aggregates characteristic of authentic VIBs, implying that the C terminus must be exposed to allow correct VIB formation. Previous structural studies suggesting a helical oligomeric structure for NS2 aggregates in which the C terminus is involved in the intermolecular interactions may help explain the necessity for the C terminus exposure, as well as the lack of aggregated VIB-like structures observed in cells transfected with pNS2-V5 [23].

Confocal microscopy, combined with data from NS2 expression studies in uninfected cells, provides evidence that NS2 can induce cell cycle arrest. In non-mitotic infected cells the NS2 appears to be associated with microtubules, consistent with interactions with a microtubule associated protein. However, the distribution of NS2 labelling in mitotic cells was diffuse, as opposed to VIB-like. This may relate to the phosphorylation status of NS2 protein, as only phosphorylated NS2 has been shown to aggregate to form VIB-like structures [21]. VIBs were observed in BTV infected cells undergoing mitosis, reflecting the more complex nature of authentic VIBs containing many other protein and RNA components, in addition to NS2.

Although infection of BHK-21 cells with the BTV-16 vaccine strain appeared to result in the greatest number of cells to undergo mitotic arrest, this effect was not strain-specific, as it was also observed with the BTV-1 reference and UK BTV-8 field strains. BHK-21 cells are highly permissive to BTV and may be inherently more sensitive to interference in the mechanism of mitosis [39]. However, this effect was also observed in Vero and BPAEC cells suggesting that it may be a more generalised feature of BTV infection/replication. It is also of note that different strains of BTV replicate at differing rates in different cell lines, for example BTV-8 (not cell culture adapted) replicates slower when compared to BTV-1 in BHK-21 cells [1] and thus the phenomena observed so dramatically during BTV-16v (cell culture adapted) infections may occur at a later time point. Further studies are needed to investigate the impact of BTV proteins/replication on the division of insect cells and more biologically relevant primary cell lines. However, the library of antibodies available for specific proteins with which to investigate this phenomenon is greatly reduced for ruminant and insect species.

The induction of cell arrest during mitosis by BTV is clearly different to the G2/S phase block induced by Reovirus [40]. However, the data described here for BTV relate well to a study by Hu *et al.*[41] using cancer cell lines. The authors found that BTV-10 was cytotoxic in cancer cells and induced a sub-G1 cell cycle arrest [41].

Infection with Bluetongue virus or expression of BTV specific proteins was shown to grossly affect two closely interrelated aspects of the mitotic process in infected cells. Firstly, it disrupts the formation of mitotic spindles, and secondly it appears to block or disrupt the interaction between the microtubules and chromosomes.

There are few reports of cell cycle modulation caused by members of the family *Reoviridae*. The T3D and T3A strains of orthoreovirus both inhibit cell proliferation by inducing a G2/M phase block via the viral attachment protein signals [40]. However, there are many examples of other viruses altering the cell cycle at different stages, favouring their replication. Examples of viruses that modulate the cell cycle include human parvovirus B19, a small DNA virus which induces cell cycle arrest at the G2/M phase by inhibiting the nuclear import of cyclin B1 [42]. Human cytomegalovirus (CMV) blocks cells entering S phase from G1, thus biasing the cellular environment towards viral replication [43], whereas Varicella Zoster Virus (VZV) ORF 12 has been found to regulate the cell cycle via the PI3K/Akt pathway [44]. Among the RNA viruses, hepatitis C virus arrests cell cycle progression at the G2/M boundary, resulting in caspase activation [45,46], and Influenza A virus NS1 protein induces a G0/G1 block by restricting the expression and activity of RhoA [47,48]. There are also reports of insect viruses causing cell cycle arrest, including *Autographa californica* nucleopolyhedrovirus (AcMNPV) (a large DNA virus of the family *Baculoviridae*) which arrests Sf9 insect cells in the G2/M phase [49]. Similarly, *Helicoverpa armigera* single-nucelocapsid nucleopolyhedrovirus (HaSNPV), causes a G2/M phase arrest in Hz-AM1 cells due to an accumulation of cyclin B1, in a manner reminiscent of parvoviruses [50].

Cell cycle arrest can be beneficial to the virus, contributing to shut-off of host cell DNA and RNA synthesis, and consequently the synthesis of host proteins, as well as causing rounding up of the cell, a cytopathic-effect frequently observed in BTV infected cells. These effects may also contribute directly to the disease pathology observed in infected animals. However, the molecular mechanisms by which most viruses modulate the cell cycle remain unclear.

In this study we found the centrosome to be disrupted in BTV-infected cells that are not undergoing mitosis. The fact that aberrant mitotic cells were observed suggests that the observed disruption may not prevent all cells from entering mitosis. However, this disruption of the centrosome in interphase cells may suggest that other stages of the cell cycle are also affected. The co-localisation of NS1 with the centrosome and the surrounding region within the cell, suggests that it could be partially responsible for the disruption of the centrosome and induction of cell cycle defects. It is possible that the components of the centriole are simply split apart, resulting in the presence of supernumary procentrioles, each in association with NS1, subsequently leading to multiple MTOCs and spindles. Further studies, including FACS analysis of synchronised cells and the expression of NS1 in isolation, are required to investigate this possibility.

The utilisation of microtubules represents an obvious approach to the challenge of shuttling viral components and/or virions throughout the cell, whether towards sites of replication, such as VIBs, or to the cell surface in preparation for release, and the host cell cytoskeleton has been shown to be fundamental at various stages in the lifecycle of numerous RNA and DNA viruses [51-57]. Our observations of NS2 associated with α-tubulin supports the hypothesis that movement along microtubules represents a potential mechanism by which NS2 could move towards the nucleus and aggregate into larger VIBs. In turn, disruptions as a result of this association may also contribute to the abnormal mitosis observed during BTV infection.

The kinetochore is a complex structure which mediates interactions between the chromosomes and microtubules [58]. Our data indicate that BTV infection does not inhibit microtubule growth, but does disrupt microtubule-chromosome interactions, resulting in a failure of the chromosomes to dock correctly via the kinetochore onto the polymerising microtubules of the mitotic spindle. NS2 appears to co-localise with the kinetochores, and this may block kinetochore attachment to the microtubule ends, contributing to the abnormal chromosomal distributions (as observed). If microtubules fail to bind to the kinetochore, the spindle checkpoint, which requires every kinetochore to be attached in a bipolar manner with equal force towards each spindle pole, would clearly fail [59,60].

These data, combined with evidence of increased apoptosis, suggest that aberrant mitosis in BTV infected cells may represent an example of ‘mitotic catastrophe’ [61]. However, it is also possible that, at least for a proportion of the arrested cells, mitotic slippage occurs, allowing the spindle checkpoint to be bypassed [62], as evidenced here by the failure of cytokinesis and the subsequent observation of binucleated cells.

A combination of mitotic slippage and multipolar spindles is consistent with our data indicating that cell division is prolonged in the presence of BTV NS2 and the observed centrosome disruption. It is conceivable that binucleation may result in aneuploidy whereby the cellular complement of chromosomes is altered from the norm. Aneuploidy can result in multiple outcomes, in particular cancer [29].

The identity of the protein that NS2 might bind to when blocking the kinetochore is unclear and represents a future direction for study, although the dynein/dynactin complex represents a possible candidate. Dynein forms a key interaction with the kinetochore [63] and several viruses including pseudorabies virus, poliovirus, hantaan virus, herpes simplex virus type 1 and hepatitis E virus, have all been shown to utilise dynein to interact with the cellular transport machinery [64-69]. The human papillomavirus protein E7, has been implicated in induction of aberrant mitotic events by interacting with the nuclear mitotic apparatus protein 1, resulting in delocalisation of dynein [70]. The tobacco mosaic virus ‘movement-protein’ has also been shown to reorganise microtubules and disrupt the centrosome, although its impact upon mitosis was not investigated [71]. Alternative proteins that might bind to NS2 to block the kinetochore include kinesins and microtubule end binding proteins [34,72].

In addition to the identification of a binding partner for NS2, the exact mechanisms by which the aberrant mitoses arise have yet to be deduced. The cell cycle is carefully regulated by a multitude of proteins, for example members of the cyclin family and p53. Further work investigating the points at which disregulation occurs to result in aberrant mitoses will be illuminating both in the field of BTV and, potentially, to the topic of mitosis in general.

## Conclusions

The importance of mitotic arrest during the BTV replication cycle is uncertain, particularly in light of the fact that our observation has not previously been reported. BTV replicates efficiently in the absence of cell division, and endothelial cells, which represent one of the targets for BTV infection in the ruminant host, divide slowly. The arrest of mitosis that is described here could represent a consequence of the virus utilising a microtubule interacting protein that is critical for mammalian cell division, to enhance its replication efficiency. It nevertheless remains an interesting observation and, based upon our initial observations in BHK-21 cells, the involvement of BTV with mitotic processes can be investigated more extensively in other biologically relevant cells. In addition, it has previously been suggested that cells could be specifically targeted using BTV as part of cancer therapy [73]. Studies with BTV may also provide a further understanding of mitotic processes and, more specifically, the cellular response when this process is perturbed or disrupted.

## Materials and methods

### Cell culture

Cells were obtained from the European Collection of Cell Cultures (ECACC). Baby hamster kidney-21 (BHK-21) cells were maintained in Glasgow’s modified Eagle’s medium (GMEM, Invitrogen) containing 2 mM L-glutamine and supplemented with 10% v/v fetal bovine serum (FBS), 5% v/v tryptose phosphate broth (TPB), 100 U penicillin ml^-1^ and 100 μg streptomycin ml^-1^ (p/s, Sigma-Aldrich). Vero (African green monkey kidney) cells were maintained in Dulbecco’s modified Eagle’s medium (DMEM) containing HEPES and supplemented with 10% v/v FBS, 2 mM L-glutamine, 2.5 μg Amphotericin B ml^-1^ and p/s. Bovine pulmonary artery endothelial (BPAEC) cells were maintained in DMEM containing HEPES and supplemented with 15% v/v FBS, 2 mM L-glutamine and p/s. For confocal microscopy, 1 × 10^4^ cells were plated in 24 well plates containing 13 mm diameter glass coverslips (Agar) 24 hours prior to infection or transfection.

The mCherry α-tubulin expression vector was a kind gift from Vic Small (Institute of Molecular Biotechnology GmbH, Vienna, Austria). This vector was transfected into HeLA cells and individual clones selected. Expression of mCherry α-tubulin was stably maintained with 750 μg/ml^-1^ Genetecin G418 in a culture media consisting of DMEM containing Glutamax, 10% v/v FBS and p/s.

All cells were maintained at 37°C in 5% CO_2_.

### Viruses

The majority of experiments in these studies were performed using the South African BTV-16 vaccine strain (BTV-16v: Orbivirus Reference Collection (ORC) number: RSAvvvv/16) which, despite an extensive *in vitro* passage history, has failed to become attenuated in sheep [74]. The South African BTV-1 reference strain (RSArrrr/01) and a BTV-8 field strain from the 2007 BTV outbreak in the United Kingdom (UKG2008/34) were also used. Details of all of the viruses are available from ORC, The Pirbright Institute (http://www.reoviridae.org/dsRNA_virus_proteins/ReoID/BTV-isolates.htm). Virus stocks were grown in BHK-21 cells by infecting monolayers at a low multiplicity of infection (MOI, ~ 0.001) and incubating the infections until cytopathic effect (CPE) was advanced (approximately 24–48 hours post infection). The cell culture supernatant fraction was clarified by centrifugation and stored at 4°C. Viruses were titrated using standard plaque assay methods in BHK-21 cells. Virus infections for downstream analysis were performed at high MOI (~10 pfu/cell) in order to obtain a uniform population of cells at an identical point in the BTV replication cycle.

### Cloning

The BTV Seg-8 open reading frame (ORF) was amplified and cloned into plasmid expression vectors to allow the expression of NS2 in isolation in mammalian cells. Briefly, RNA was first extracted from the cellular fraction of BTV infected BHK-21 cells using TRIzol (Invitrogen) and reverse transcribed using Moloney murine leukaemia virus reverse transcriptase (Invitrogen) according to the manufacturer’s instructions. The NS2 open reading frame of BTV-16v was amplified using KOD Hotstart DNA polymerase (Novagen, Merck) and cloned into pcDNA3.1 V5/HIS-TOPO (Invitrogen), resulting in pNS2-V5. The coding sequence of BTV-1 NS2 was amplified using primers containing BsrG1 and NotI restriction sites. The purified PCR products were digested with BsrG1 and NotI and cloned into pEFGP-N1 (Clontech) digested using these enzymes, resulting in pEGFP-NS2. When transfected into mammalian cells, the pNS2-V5 plasmid expresses NS2 with C-terminal V5 and HIS epitopes, whereas pEGFP-NS2 expresses NS2 with enhanced green fluorescent protein (EGFP) fused to the amino terminus.

### Transfection of plasmids into mammalian cells

Plasmid transfections for confocal analysis were performed using FuGene HD (Promega) according to the manufacturer’s instructions. Transfection complexes were generated by diluting 2 μg of plasmid DNA in 100 μl of OptiMEM serum free media (Gibco). 5 μl of FuGene HD was added to the diluted DNA, vortexed briefly to mix, and incubated for 15 min at room temperature. Cell transfections were performed in 24 well plates using 25 μl of the complexes added dropwise to 0.5 ml of media in the well. The plate was rocked to distribute the complexes before incubation at 37°C in 5% CO_2._ The complexes were not removed until fixation of the cells at 24 hours post-transfection.

HeLa-mCherry tubulin cells were grown in 35 mm glass bottomed dishes (Ibidi Hi-Q4) to reach 40 - 50% confluency by the day of transfection and then transiently transfected with pEGFP-N1 or pEGFP-NS2 using GeneJuice® (Novagen). 100 μl of serum free medium was mixed with 3 μl GeneJuice® reagent by vortexing. After 5 min incubation at room temperature, 1 μg of plasmid DNA was added to the GeneJuice®/medium mixture, gently mixed and incubated for a further 20 min at room temperature. The medium on the HeLa-mCherry tubulin cells was replaced and the transfection complex added dropwise. The cells were incubated for 24 h and examined for EGFP-fluorescence to determine transfection efficiency, before live cell imaging.

### Cell fixation for confocal microscopy

Cells on coverslips were fixed by transferring the coverslips into a new 24 well plate containing room temperature 4% (w/v) paraformaldehyde in phosphate buffered saline (PBS), or ice cold 100% methanol depending upon the antibody to be used in downstream labelling. The cells were allowed to fix for 40 min at room temperature (paraformaldehyde) or 5 minutes at −20°C (methanol) before washing with PBS.

### Antibody labelling and coverslip mounting

All incubations were performed at room temperature. Cells were permeabilised for 15 min in 0.1% v/v Triton X-100 in PBS, washed once in PBS, and incubated for 0.5 h in blocking buffer (PBS containing 0.5% w/v bovine serum albumin (BSA)). The coverslips were incubated with primary antibodies in blocking buffer for 1 h, washed three times in PBS, and incubated for 1 h with species-specific goat secondary antibodies conjugated to Alexa Fluor-568 or Alexa Fluor-488 (Molecular Probes, Invitrogen Paisley, United Kingdom) diluted 1:200 in blocking buffer. Isotype-specific Alexa Fluor conjugated secondary antibodies were used in combination with primary mouse monoclonal antibodies. After incubation with the antibodies, the coverslips were washed three times in PBS, incubated for 10 min in 1 × 4′6′-diamidino-2-phenylindole (DAPI), rinsed in deionised water and mounted onto glass slides using VECTASHIELD®. The coverslips were then sealed onto the slide using clear nail varnish and stored in the dark at 4°C until examination by confocal microscopy. All antibodies had been previously characterised and found to be suitable for use in confocal microscopy. Orab 1 is a polyclonal antibody generated in rabbits following inoculation with *in vitro* expressed and purified BTV-1 NS2. ‘No primary antibody’ controls were performed on with/without treatment samples, to confirm the specificity of the antibody. All experiments involving infections included uninfected controls. Experiments incorporating plasmid transfections, also included ‘reagent only’ (no plasmid DNA) and ‘empty vector’ controls.

Confocal microscopy of fixed and immunolabeled coverslips was carried out using a Leica SP2 laser scanning confocal microscope. Captured images were adjusted in Adobe Photoshop CS2 version 9.0.

### Live cell imaging

Live cell imaging was performed using a Nikon BioStation IM CELL-S1 microscopy system (Nikon UK Ltd, Surrey, UK). On the day of imaging the medium was removed from the cells and replaced with CO_2_-independent medium (Invitrogen) supplemented with 10% FBS and 4 mM glutamine. The imaging dish was placed in the BioStation and multiple fields of views were selected for time-lapse imaging during system equilibration. Set up and data processing were carried out according to Morrison *et al.*[75]. Three individual images (phase contrast, EGFP and mCherry) were captured once every 3 min for a period of 24 h for all selected fields of view. For each condition, (untransfected, pEGFP only plasmid, or pEGFP-NS2) at least 50 mitotic cells were identified in the selected fields and movies were examined using QuickTime Player. Mitosis was defined by the onset of nuclear envelope breakdown until midbody formation, indicating initiation of cytokinesis. Movies were analysed for length of time spent in mitosis, mitotic failure rate (cells that did not finish mitosis and appeared to undergo apoptosis) and binucleation events (cells which were binucleated following mitosis). Data were analysed and p values generated using Prism 6 software.

## Competing interests

The authors declare that they have no competing interests.

## Authors’ contributions

Conceived and designed experiments: AS, AB-R, EEM, PPCM, PM. Performed experiments: AS, AB-R, JS, PM. Analysed data: AS, AB-R, EEM, OA, PM. Contributed reagents: JB, NR-S. Wrote the manuscript: AS, AB-R, EEM, PPCM, PM. All authors read and approved the final manuscript.
